# Increased IL-15 Production and Accumulation of Highly Differentiated CD8^+^ Effector/Memory T Cells in the Bone Marrow of Persons with Cytomegalovirus

**DOI:** 10.3389/fimmu.2017.00715

**Published:** 2017-06-19

**Authors:** Luca Pangrazzi, Erin Naismith, Andreas Meryk, Michael Keller, Brigitte Jenewein, Klemens Trieb, Beatrix Grubeck-Loebenstein

**Affiliations:** ^1^Department of Immunology, Institute for Biomedical Aging Research, University of Innsbruck, Innsbruck, Austria; ^2^Department of Orthopedic Surgery, Hospital Wels-Grieskirchen, Wels, Austria

**Keywords:** bone marrow, cytomegalovirus, aging, immunosenescence, senescence

## Abstract

Cytomegalovirus (CMV) has been described as a contributor to immunosenescence, thus exacerbating age-related diseases. In persons with latent CMV infection, the CD8^+^ T cell compartment is irreversibly changed, leading to the accumulation of highly differentiated virus-specific CD8^+^ T cells in the peripheral blood. The bone marrow (BM) has been shown to play a major role in the long-term survival of antigen-experienced T cells. Effector CD8^+^ T cells are preferentially maintained by the cytokine IL-15, the expression of which increases in old age. However, the impact of CMV on the phenotype of effector CD8^+^ T cells and on the production of T cell survival molecules in the BM is not yet known. We now show, using BM samples obtained from persons who underwent hip replacement surgery because of osteoarthrosis, that senescent CD8^+^ T_EMRA_ cells with a bright expression of CD45RA and a high responsiveness to IL-15 accumulate in the BM of CMV-infected persons. A negative correlation was found between CMV antibody (Ab) titers in the serum and the expression of CD28 and IL-7Rα in CD8^+^ ${\text{T}}_{\text{EMRA}}^{\text{bright}}$ cells. Increased IL-15 mRNA levels were observed in the BM of CMV^+^ compared to CMV^−^ persons, being particularly high in old seropositive individuals. In summary, our results indicate that a BM environment rich in IL-15 may play an important role in the maintenance of highly differentiated CD8^+^ T cells generated after CMV infection.

## Introduction

Aging is associated with a decline of immune function, a condition known as immunosenescence, which reduces the capability to fight infections, thus contributing to age-related diseases. Due to thymic involution, the generation of new naïve T cells diminishes with age (1, 2). In parallel, more differentiated T cells accumulate in the elderly, particularly in the bone marrow (BM) (3, 4). Recently, the important role of the BM in producing the T cell survival factors IL-15 and IL-7, which are necessary for the long-term maintenance of effector/memory T cells, has been documented (5–8). In particular, IL-15 has been shown to be important for the preservation of highly differentiated CD8^+^ effector T cells (9–11). In old age, a proinflammatory BM environment promotes the accumulation of IL-15, which may lead to increased numbers of highly differentiated T cells as a consequence (12).

A major contributor to immunosenescence is cytomegalovirus (CMV), a lifelong-persisting herpes virus present in 60–100% of adult individuals depending on the cohort (13, 14). CMV infection has been linked to increased CRP levels in the blood and diseases with an inflammatory component such as cardiovascular disease and cancer (15–18). Even in healthy carriers, CMV-specific T cells expand over time leading to memory inflation (19–21). Although inflation of CD4^+^ T cells has also been observed, virus-specific effector/memory CD8^+^ T cells accumulate in the peripheral blood (PB) at higher frequencies (19, 22, 23). In addition, CMV seropositivity has been associated with an inverted CD4:CD8 ratio in the blood in old age (24). In the elderly, the majority of effector/memory CMV-specific CD8^+^ T cells has been shown to re-express CD45RA, acquiring the typical feature of terminally differentiated cells (25–27). Although the CD8^+^ T cell phenotype in CMV^+^ persons has been extensively described in the PB, the impact of CMV on BM CD8^+^ T cells has only been partially investigated so far.

In the present study, the phenotypes of effector CD8^+^ T cell subsets in the BM of CMV^−^ and CMV^+^ persons were compared. A population of CD8^+^ T_EMRA_ cells with a bright expression of CD45RA, low levels of CD28, and expressing the senescence marker killer cell lectin-like receptor G1 (KLRG-1) expanded in CMV^+^ persons. While the responsiveness of these BM CD8^+^ ${\text{T}}_{\text{EMRA}}^{\text{bright}}$ cells to IL-15 was high, the expression of IL-7Rα was reduced. In addition, CMV antibody (Ab) titers in the serum correlated negatively with the expression of CD28 and IL-7Rα in CD8^+^ ${\text{T}}_{\text{EMRA}}^{\text{bright}}$ cells and positively with a ratio between CD122 (IL-2/IL-15Rβ) and IL-7Rα^+^ cells. Increased IL-15 mRNA expression and more interactions between CD8^+^ T cells and IL-15-producing cells were found in the BM of CMV^+^ persons. Our results show that, in CMV^+^ persons, IL-15 may contribute to the accumulation and the survival of senescent CD8^+^ T_EMRA_ cells in the BM.

## Materials and Methods

### Study Subjects

Samples were obtained from systemically healthy individuals who did not receive immunomodulatory drugs or suffer from diseases known to influence the immune system, such as autoimmune diseases and cancer. None of them was frail or had symptoms of cognitive impairments. In all patients, the indication for surgery was osteorarthrosis. Further information about the donors included in the study is summarized in Table 1.

**Table 1:** Demographic data of the donors included in the study, divided into cytomegalovirus (CMV)^−^ and CMV^+^ groups.

	CMV^−^	CMV^+^
*N*	33	37
Sex	14F, 19M	20F, 17M
Age range (years)	43–87	32–87
Mean age (years)	66 ± 10	70 ± 11
Body weight	76.2 ± 18	82.3 ± 11
Hip fracture (%)	0	0

### Sample Collection and Preparation

Hip replacement surgery was performed and bone from the femur shaft was harvested. A biopsy of *substantia spongiosa osseum*, which would otherwise have been discarded, was used to isolate BM mononuclear cells (BMMCs). BM biopsies were fragmented, washed once with complete RPMI medium (RPMI 1640 supplemented with 10% FCS, 100 U/ml penicillin, and 100 μg/ml streptomycin; Invitrogen) and treated with purified collagenase (CLSPA, Worthington Biochemical; 20 U/ml in complete RPMI medium) for 1 h at 37°C. BM biopsies were then centrifuged and BMMCs purified by density gradient centrifugation (Ficoll-Hypaque). Purification of PB mononuclear cells (PBMCs) from heparinized blood was also performed by density gradient centrifugation.

### Isolation of RNA and Quantitative RT-PCR

RNA was isolated from purified BMMCs using the RNeasy Plus mini kit (Qiagen). First-strand cDNA synthesis was performed using a Reverse Transcription system (Promega). Quantitative RT-PCR experiments were performed using the LightCycler 480 System (Roche Diagnostics), 2X SYBR Green 1 Master (Roche Diagnostics), and β-actin as housekeeping gene for relative quantification of effector/memory cell survival factors. Sequence-specific oligonucleotide primers were designed using Primer3 software (25) and synthesized by MWG Biotech (Ebersberg, Germany). The following primers were used: IL-15FW 5′-ATTTTGGGCTGTTTCAGTGC-3′, IL-15RW 5′-TTACTTTGCAACTGGGGTGA-3′, IL-7FW 5′-GTAGCAATTGCCTGAATAATG-3′, IL-7RW 5′-GTTGTGCCTTCTGAAACT-3′.

### Flow Cytometric Analysis

Immunofluorescence surface staining was performed by adding a panel of directly conjugated Abs to BMMCs. After surface staining, cells were permeabilized using the Cytofix/Cytoperm kit (BD Pharmingen), and incubated with intracellular Abs. Labeled cells were measured using a FACSCanto II (BD Biosciences). Data were analyzed using Flowjo software. The following labeled Abs were used: IL-7Rα-PE (hIL-7Rα-M21), CD8-PeCy7 (RPA-T8), CCR7-FITC (150503), and CD28 BV421 (CD28.2) purchased from BD, CD122 (IL-2/IL-15Rβ)-APC (TU-27), CD45RA-PerCp (HI100), and KLRG-1-PeCy7 (2F1/KLRG1) purchased from Biolegend, CD3-APC-eFluor 780 (SK7) purchased from eBioscience.

### Responsiveness to BM CD8^+^ T Cell Survival Factors

The responsiveness of CD8^+^ T cell subsets to IL-15 and IL-7 was measured by quantifying the cells expressing the receptors CD122 (IL-2/IL-15Rβ) and IL-7Rα, respectively (26–28).

### Immunofluorescence Analysis of BM Biopsies

Immunofluorescence analysis of BM sections was performed, as described by Herndler-Brandstetter et al. (8). Formalin-fixed, paraffin-embedded 4-µm BM sections were deparaffinized in xylene and re-hydratated in ethanol. The slides were boiled in 0.01 M citrate buffer (pH 6) for 16 min in the microwave for epitope retrieval and allowed to cool for about 1 h at room temperature. Slides were blocked with 3% skim milk in TBS/Tween for 20 min at room temperature. Rabbit anti-IL-15 (1:200; ab55276; Abcam) and mouse anti-CD8 (1:50; C8/144B; Dako) Abs were incubated overnight at 4°C. After washing, the slides were incubated for 1 h at 4°C with biotinylated swine anti-rabbit Ab (1:300; E0431; Dako) and a goat anti-mouse Alexa Fluor 546 Ab (1:300; A11018; Molecular Probes). Following washing steps with TBS/0.1% Tween, the BM sections were stained with a streptavidine-Alexa Fluor 488 Ab (1:500; S11223; Molecular Probes) for 30 min at 4°C. The stained slides were analyzed using confocal microscopy with an m-Radiance confocal scanning system (Bio-Rad) attached to a Zeiss Axiophot microscope (Carl Zeiss). For the quantitative analysis of CD8^+^ T cells in close contact with IL-15–producing cells in the BM, pictures from different areas of the BM sections were analyzed. In total, 800–1,000 CD8^+^ T cells were analyzed from each donor to calculate the percentage of contact with IL-15^+^ cells.

### Determination of CMV Seropositivity

Antibodies against CMV were determined in the serum of the donors included in the study using a commercially available ELISA Kit (Siemens).

### Statistical Analysis

The data obtained in the study follow a non-parametric distribution. Statistical significance was assessed by Spearman correlation analysis, Mann–Whitney test and Wilcoxon matched pairs test. A *p-*value of less than 0.05 was considered as significant.

### Study Approval

The study was approved by the Ethics Committees of the “Klinikum Wels-Grieskirchen” (Austria). Written informed consent was received from participants prior to their inclusion in the study.

## Results

### CD8 ${\text{T}}_{\text{EMRA}}^{\text{bright}}$ Cells, which Are KLRG-1^+^ and Frequently Lack CD28, Increase in the BM of CMV^+^ Persons

**Figure 1:**
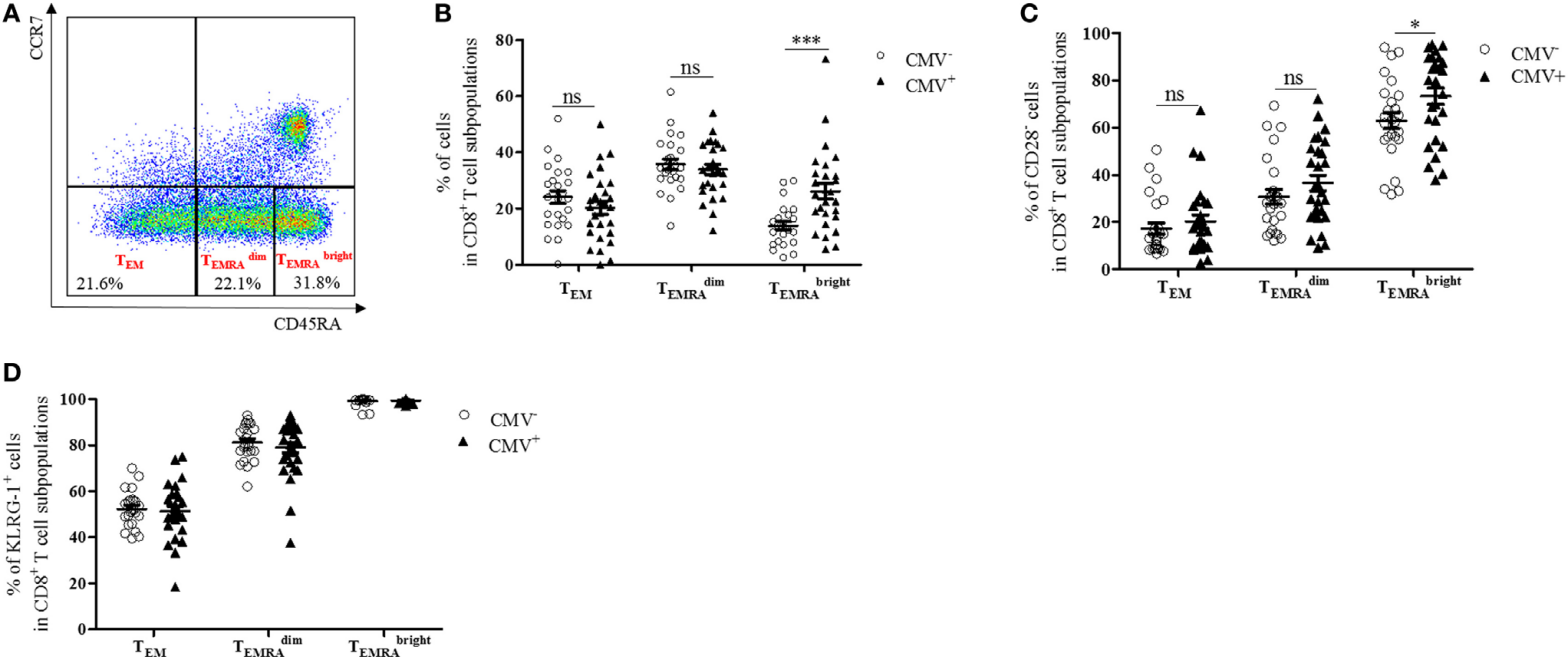
CD8^+^ ${\text{T}}_{\text{EMRA}}^{\text{bright}}$ cells with a senescent phenotype, which typically lack CD28 increase in the bone marrow of cytomegalovirus (CMV)^+^ persons. **(A)** FACS plot showing gating strategies for T_EM_, ${\text{T}}_{\text{EMRA}}^{\text{dim}}$, and ${\text{T}}_{\text{EMRA}}^{\text{bright}}$ subsets in CD8^+^ T cells. In CCR7^−^ CD8^+^ T cells, a population of CD45RA^−^ (T_EM_) cells, one with an intermediate $\left({\text{T}}_{\text{EMRA}}^{\text{dim}}\right)$ and one with a high expression of CD45RA $\left({\text{T}}_{\text{EMRA}}^{\text{bright}}\right)$ were defined. **(B)** Percentages of T_EM_, ${\text{T}}_{\text{EMRA}}^{\text{dim}}$, and ${\text{T}}_{\text{EMRA}}^{\text{bright}}$ subsets in CD8^+^ T cells (=100%) in CMV^−^ (dots) and CMV^+^ (triangles) persons. Mann–Whitney test, ***p < 0.001. **(C)** Percentages of CD28^−^ T cells in CD8^+^ T_EM_, ${\text{T}}_{\text{EMRA}}^{\text{dim}}$, and ${\text{T}}_{\text{EMRA}}^{\text{bright}}$ subsets in CMV^−^ and CMV^+^ persons. Mann–Whitney test, *p = 0.05. **(D)** Percentages of KLRG-1^+^ cells in CD8^+^ T_EM_, ${\text{T}}_{\text{EMRA}}^{\text{dim}}$, and ${\text{T}}_{\text{EMRA}}^{\text{bright}}$ cells in CMV^−^ and CMV^+^ persons. *N* = 26 (CMV^−^ group) and *N* = 28 (CMV^+^ group).

CD8^+^ T cells with a CD45RA^+^ CCR7^−^ T_EMRA_ phenotype have been shown to accumulate in the blood after CMV infection (29, 30). To assess whether T_EMRA_ cells are also enriched in the BM from CMV^+^ persons, we measured the levels of BM CD8^+^ T_EMRA_ cells in CMV^−^ and CMV^+^ persons (Figure 1). CD8^+^ T_EMRA_ cells were gated, as indicated in Figure 1A. Within the CD8^+^ CCR7^−^ T cell population, a subpopulation with an intermediate $\left({\text{T}}_{\text{EMRA}}^{\text{dim}}\right)$ and one with a bright $\left({\text{T}}_{\text{EMRA}}^{\text{bright}}\right)$ expression of CD45RA and a subset, which does not express CD45RA (T_EM_) were defined. Higher percentages of both ${\text{T}}_{\text{EMRA}}^{\text{dim}}$ and ${\text{T}}_{\text{EMRA}}^{\text{bright}}$ cells were found in BMMCs compared to PBMCs (${\text{T}}_{\text{EMRA}}^{\text{dim}}$ *p* = 0.001; ${\text{T}}_{\text{EMRA}}^{\text{bright}}$ *p* = 0.04, data not shown). While the size of the CD8^+^ T_EM_ and the CD8^+^ ${\text{T}}_{\text{EMRA}}^{\text{dim}}$ populations in the BM was similar in CMV^−^ and CMV^+^ persons, the percentage of CD8^+^ ${\text{T}}_{\text{EMRA}}^{\text{bright}}$ cells was higher in CMV^+^ persons (Figure 1B). No differences were observed in the numbers of CD8^+^ T_EM_, ${\text{T}}_{\text{EMRA}}^{\text{dim}}$, and ${\text{T}}_{\text{EMRA}}^{\text{bright}}$ cells when we compared younger (≤70 years) and older (>70 years) donors in both the CMV^−^ and the CMV^+^ group (n.s., data not shown).

CD8^+^ T_EMRA_ cells have been described to downregulate CD28 and to express senescence markers in the PB (31). CD8^+^ CD28^−^ T cells have also been observed to accumulate in the BM in old age (12, 32). To assess whether CMV affects the phenotype of CD8^+^ subsets in the BM, we analyzed CD8^+^ CD28^−^ T cells in the BM of CMV^−^ and CMV^+^ persons (Figure 1C). Increased frequencies of CD8^+^ CD28^−^ ${\text{T}}_{\text{EMRA}}^{\text{bright}}$ cells were found in CMV^+^ compared with CMV^−^ persons, while no differences between the two groups were observed in CD8^+^ T_EM_ and CD8^+^ ${\text{T}}_{\text{EMRA}}^{\text{dim}}$ cells. While CD8^+^ CD28^−^ ${\text{T}}_{\text{EMRA}}^{\text{bright}}$ T cells correlated positively with age in CMV^−^ persons, no age-related changes were observed for CD28^−^ ${\text{T}}_{\text{EMRA}}^{\text{bright}}$ cells in CMV^+^ persons and for T_EM_ and ${\text{T}}_{\text{EMRA}}^{\text{dim}}$ cells in both the CMV^+^ and the CMV^−^ group (Figure S1 in Supplementary Material).

We then analyzed the expression of the senescence marker KLRG-1 (33) in BM CD8^+^ T cell subsets and compared the results in CMV^+^ and CMV^−^ persons (Figure 1D). The percentage of KLRG-1-expressing cells was relatively low in T_EM_, high in ${\text{T}}_{\text{EMRA}}^{\text{dim}}$, and was even higher in ${\text{T}}_{\text{EMRA}}^{\text{bright}}$ cells. No differences were observed when the CMV serostatus was considered or when younger (≤70 years) and older (>70 years) donors were compared (data not shown). These data suggest that CD8^+^ T_EMRA_ cells with a high expression of CD45RA, which frequently lack CD28 and express KLRG-1, increase in the BM of CMV^+^ persons.

### CD8^+^ ${\text{T}}_{\text{EMRA}}^{\text{bright}}$ Cells with a High Expression of CD122 and a Reduced Expression of IL-7Rα Increase in the BM of CMV^+^ Persons

**Figure 2:**
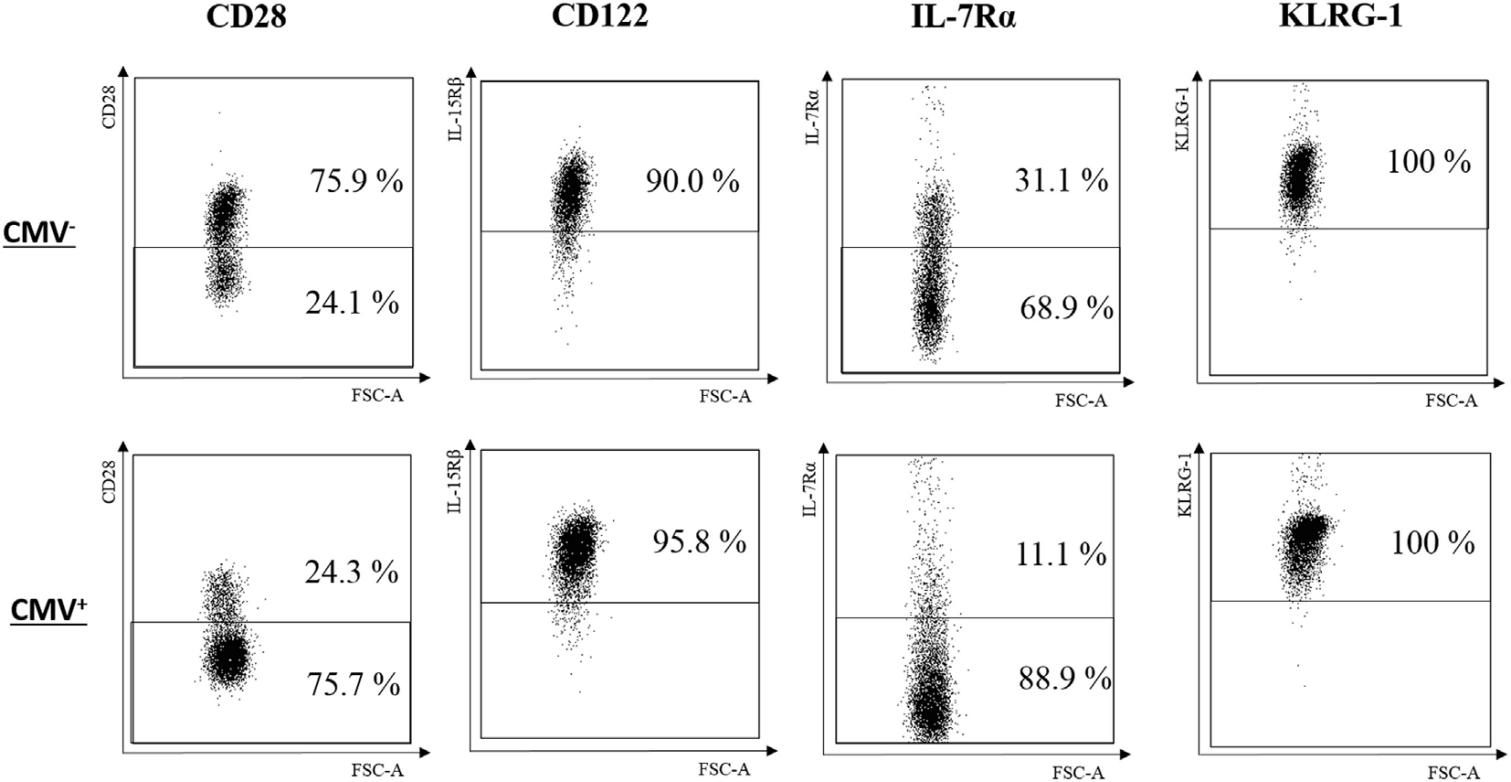
Typical phenotype of CD8^+^ ${\text{T}}_{\text{EMRA}}^{\text{bright}}$ cells in cytomegalovirus (CMV)^−^ and CMV^+^ persons. FACS plots for CD28, CD122, IL-7Rα, and KLRG-1 from one representative CMV^−^ (69 years) and one CMV^+^ (72 years) person are shown.

**Figure 3:**
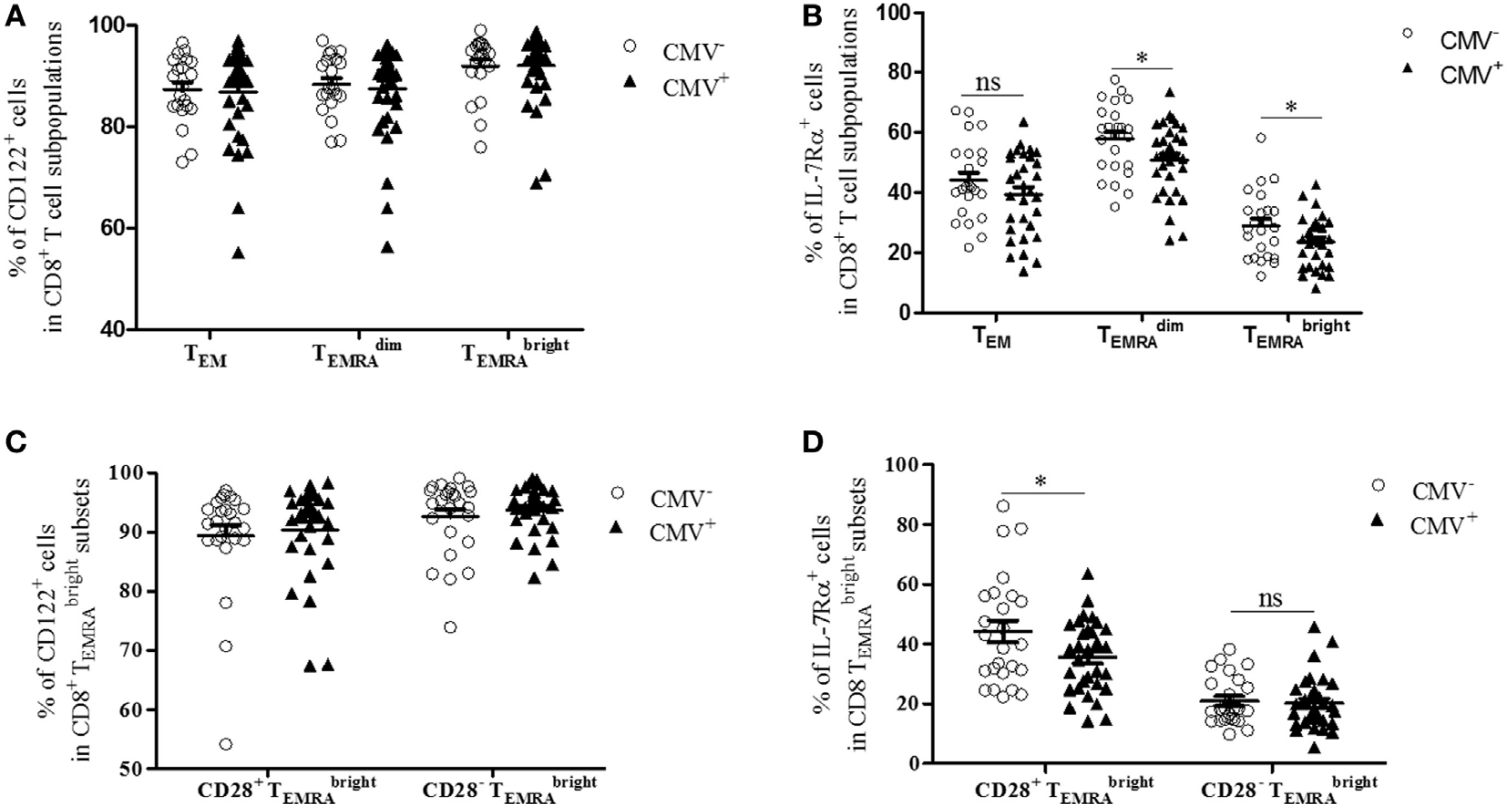
Expression of CD122 and IL-7Rα in bone marrow CD8^+^ T cell subsets from cytomegalovirus (CMV)^−^ and CMV^+^ persons. Percentages of **(A)** CD122^+^ and **(B)** IL-7Rα^+^ cells in CD8^+^ T_EM_, ${\text{T}}_{\text{EMRA}}^{\text{dim}}$, and ${\text{T}}_{\text{EMRA}}^{\text{bright}}$ subpopulations in CMV^−^ and CMV^+^ persons. Mann–Whitney test, *p < 0.05. *N* = 32 (CMV^−^ group) and *N* = 37 (CMV^+^ group). Wilcoxon-matched pairs test, CD8^+^ ${\text{T}}_{\text{EMRA}}^{\text{dim}}$ vs. CD8^+^ ${\text{T}}_{\text{EMRA}}^{\text{bright}}$ cells in the whole cohort (CMV^+^ plus CMV^−^) ***p < 0.001 (significance not indicated in the Figure). Percentages of **(C)** CD122^+^ and **(D)** IL-7Rα^+^ cells in CD8^+^ CD28^+^ and CD8^+^ CD28^−^ ${\text{T}}_{\text{EMRA}}^{\text{bright}}$ cells from CMV^−^ and CMV^+^ persons. Mann–Whitney test, *p < 0.05. *N* = 32 (CMV^−^ group) and *N* = 37 (CMV^+^ group).

IL-7 and IL-15 influence the survival and turnover of CD8^+^ T cells (34). They are of particular relevance for the interaction of T cells with stromal cells in the BM (8, 35). We, therefore, decided to study the expression of CD122 and IL-7Rα in CD8^+^ T cells. Specifically, we compared these parameters in the T_EM_, ${\text{T}}_{\text{EMRA}}^{\text{dim}}$, and ${\text{T}}_{\text{EMRA}}^{\text{bright}}$ subsets from CMV^−^ and CMV^+^ persons (Figures 2 and 3). The “typical” phenotype of a CD8^+^ ${\text{T}}_{\text{EMRA}}^{\text{bright}}$ cell in CMV^−^ and CMV^+^ donors is shown in Figure 2. In the whole cohort (CMV^+^ plus CMV^−^ persons), CD122 was expressed on a great majority of cells, in all subsets, but on almost every cell in the CD8^+^ ${\text{T}}_{\text{EMRA}}^{\text{bright}}$ population (Wilcoxon matched pairs test, *p* < 0.001 ${\text{T}}_{\text{EMRA}}^{\text{bright}}$ vs. ${\text{T}}_{\text{EMRA}}^{\text{dim}}$, Figure 3A). No differences were found between CMV^−^ and CMV^+^ persons. Interestingly, when relating the expression of CD122 with age, in CD8^+^ T cell subsets from CMV^−^ and CMV^+^ persons, we found positive correlations in CMV^+^ persons, whereas negative correlations were seen among T_EM_ and ${\text{T}}_{\text{EMRA}}^{\text{dim}}$ subsets (Figures S2A,B in Supplementary Material). No significant correlations with age were found in CD8^+^ ${\text{T}}_{\text{EMRA}}^{\text{bright}}$ cells (Figure S2C in Supplementary Material). Lower percentages of IL-7Rα^+^ cells were observed in both CD8^+^ ${\text{T}}_{\text{EMRA}}^{\text{dim}}$ and ${\text{T}}_{\text{EMRA}}^{\text{bright}}$ cells from CMV^+^ persons when compared to their CMV^−^ counterparts (Figure 3B). In CMV^−^ persons, there was a positive correlation between IL-7Rα and age in CD8^+^ T_EM_ cells, while negative correlations were seen between CD8^+^ ${\text{T}}_{\text{EMRA}}^{\text{dim}}$ and ${\text{T}}_{\text{EMRA}}^{\text{bright}}$ cells in CMV^+^ persons (Figures S2D–F in Supplementary Material).

We then compared the expression of CD122 and IL-7Rα in CD8^+^ CD28^+^ and CD8^+^ CD28^−^ ${\text{T}}_{\text{EMRA}}^{\text{bright}}$ cells (Figures 3C,D). Higher percentages of CD122^+^ cells were found in CD8^+^ CD28^−^ ${\text{T}}_{\text{EMRA}}^{\text{bright}}$ cells compared to their CD8^+^ CD28^+^ counterparts (Wilcoxon matched pairs test, *p* = 0.01 in CMV^−^ and *p* = 0.002 in CMV^+^ persons; Figure 3C). No differences were seen between CMV^−^ and CMV^+^ persons. In contrast, the percentage of IL-7Rα^+^ cells was lower in CD8^+^ CD28^−^ ${\text{T}}_{\text{EMRA}}^{\text{bright}}$ cells compared to CD8^+^ CD28^+^ ${\text{T}}_{\text{EMRA}}^{\text{bright}}$ cells (Wilcoxon matched pairs test, *p* < 0.001 in both CMV^−^ and CMV^+^ persons; Figure 3D). When comparing CMV^+^ and CMV^−^ samples, reduced numbers of IL-7Rα^+^ cells were seen in CMV^+^ persons within the CD8^+^ CD28^+^ ${\text{T}}_{\text{EMRA}}^{\text{bright}}$ subset; however, no differences were seen within the CD8^+^ CD28^−^ ${\text{T}}_{\text{EMRA}}^{\text{bright}}$ population. In summary, our findings indicate that numbers of CD8^+^ ${\text{T}}_{\text{EMRA}}^{\text{bright}}$ cells with high CD122 and/or low IL-7Rα expression increase in the BM of CMV^+^ persons. Thus, in CMV^+^ persons, the “typical” CD8^+^ ${\text{T}}_{\text{EMRA}}^{\text{bright}}$ cell is most likely CD28^−^, CD122^hi^ IL-7Rα^low^, and KLRG-1^+^; however, whether every cell in the ${\text{T}}_{\text{EMRA}}^{\text{bright}}$ subset carries the full marker pattern is not yet known and is currently being investigated. In CMV^−^ persons, the phenotype of CD8^+^ ${\text{T}}_{\text{EMRA}}^{\text{bright}}$ cells differs somewhat as these cells still express reasonably high levels of both CD28 and IL-7Rα.

### CMV Ab Titers Correlate Positively with CD28^−^ Cells and Negatively with IL-7Rα^+^ Cells in CD8^+^ ${\text{T}}_{\text{EMRA}}^{\text{bright}}$ Cells

**Figure 4:**
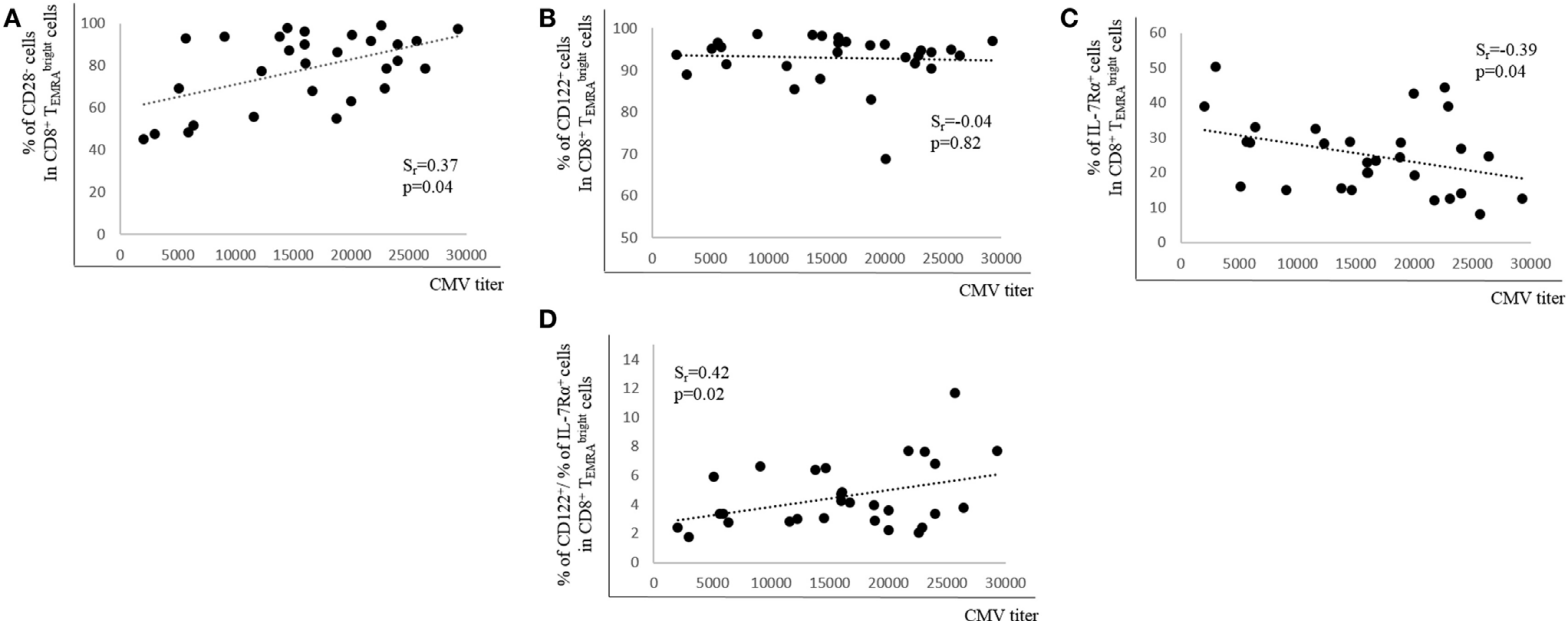
Relationship between cytomegalovirus (CMV) antibody (Ab) titers and expression of CD28, CD122, and IL-7Rα in bone marrow CD8^+^ ${\text{T}}_{\text{EMRA}}^{\text{bright}}$ T cells. Percentages of **(A)** CD28^−^, **(B)** CD122^+^, **(C)** IL-7Rα^+^ cells, and **(D)** the ratio between the percentages of CD122^+^ and of IL-7Rα^+^ cells in the CD8^+^ ${\text{T}}_{\text{EMRA}}^{\text{bright}}$ subset in relationship to CMV Ab titers in the serum are shown. Spearman coefficient (*r*_s_) and *p* values are shown in each graph (*N* = 29).

Although it is not clear which role CMV-specific Abs play in latent CMV infection, high Ab titers are connected with CMV re-activation (36). We, therefore, determined whether CMV Ab titers correlated with the percentage of CD28^+^, CD122^+^, and IL-7Rα^+^ expression in BM CD8^+^ ${\text{T}}_{\text{EMRA}}^{\text{bright}}$ cells. There was a positive correlation between Ab titers and the percentage of CD28^−^ cells in the CD8^+^ ${\text{T}}_{\text{EMRA}}^{\text{bright}}$ population, but no relationship between Ab titers and CD122-expressing cells was observed (Figures 4A,B). In contrast, when the percentage of IL-7Rα^+^ cells in the CD8^+^ ${\text{T}}_{\text{EMRA}}^{\text{bright}}$ population was assessed in relationship to CMV Ab titers, there was a negative correlation (Figure 4C). In addition, a ratio between the percentages of CD122^+^ cells and of IL-7Rα^+^ cells in the CD8^+^ ${\text{T}}_{\text{EMRA}}^{\text{bright}}$ population was calculated (% CD122^+^ cells/% IL-7Rα^+^ cells). When this ratio was correlated with CMV Ab titers, a positive correlation was found (Figure 4D). Thus, our data indicate that CMV Ab titers in the serum correlate with the expression of CD28 and IL-7Rα as well as with the ratio CD122^+^/IL-7Rα^+^ in CD8^+^ ${\text{T}}_{\text{EMRA}}^{\text{bright}}$ cells.

### IL-15 but Not IL-7 mRNA Expression in the BM Is Affected by CMV and More CD8^+^ T Cells Are in Close Proximity to IL-15-Producing Cells

**Figure 5:**
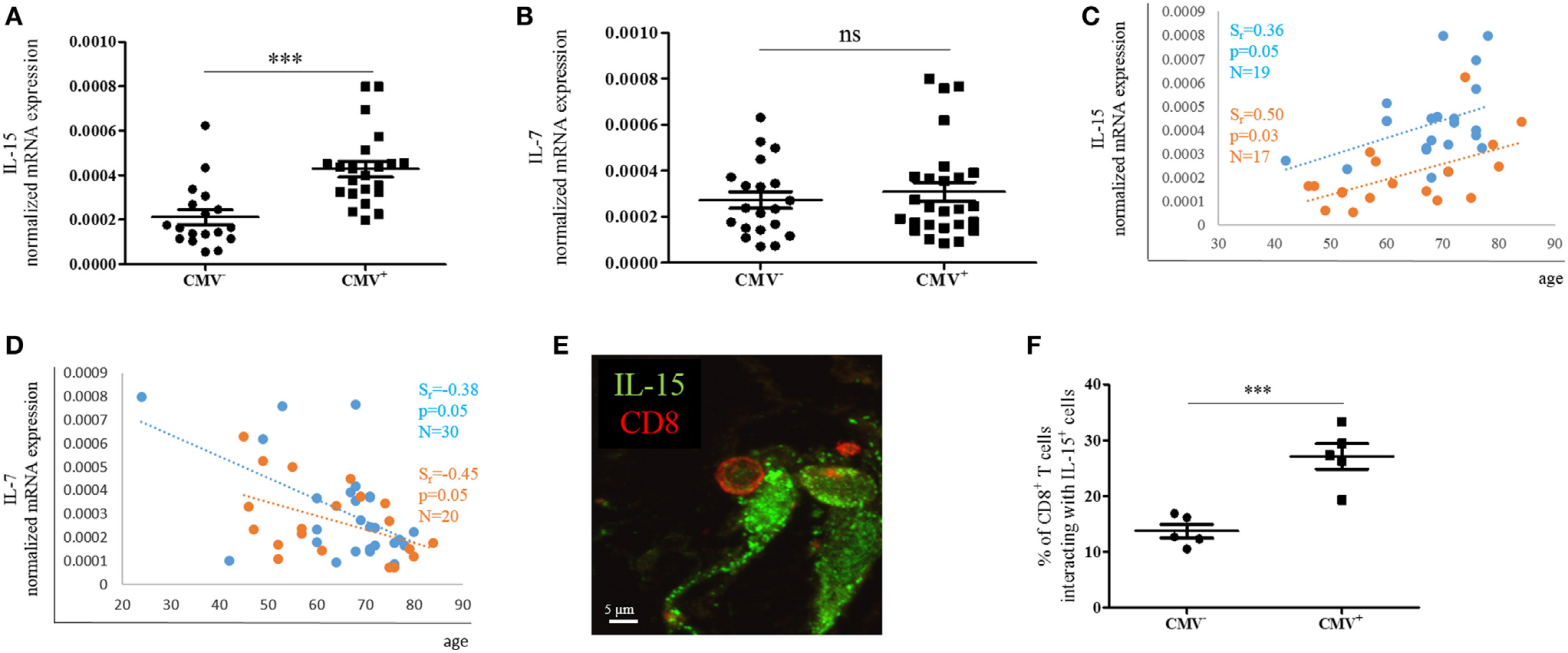
mRNA expression of IL-15 and IL-7 in bone marrow (BM) mononuclear cells from cytomegalovirus (CMV)^−^ and CMV^+^ persons. mRNA expressions of **(A)** IL-15 and **(B)** IL-7 in the CMV^−^ and CMV^+^ group. Mann–Whitney test, *p* < 0.001. The sample size is shown in the graphs in **(C,D)**. The mRNA expressions of **(C)** IL-15 and **(D)** IL-7 in CMV^−^ (orange) and CMV^+^ (blue) persons in correlation with age are shown. Spearman coefficient (*r*_s_), *p*-value, and sample size (*N*) are shown in each graph. The mRNA expression of each gene was normalized against the housekeeping gene β-actin. **(E)** Representative picture obtained after immunofluorescence staining of BM sections showing one interaction between a CD8^+^ T cell (red) and an IL-15^+^ cell (green). **(F)** Percentages of CD8^+^ T cells interacting with IL-15-producing cells in the BM of CMV^−^ and CMV^+^ persons. Mann–Whitney test, *N* = 5 for each group (***p < 0.001).

In order to assess whether the expression of effector/memory T cell survival factors in the BM differs in CMV^+^ persons, we measured the expression of IL-15 and IL-7 at the mRNA level in BMMCs from CMV^−^ and CMV^+^ persons of varying ages (Figure 5). Indeed, higher IL-15 mRNA levels were found when CMV^+^ persons were compared with their CMV^−^ counterparts (Figure 5A). IL-15 mRNA was 1.9 ± 0.1-fold higher in BMMCs from CMV^+^ compared to CMV^−^ persons. No difference in the expression of IL-7 mRNA was observed (Figure 5B). In a previous study, we demonstrated that IL-15 increased while IL-7 decreased during aging in the BM (12). We now confirm these data in CMV^−^ and CMV^+^ persons (Figures 5C,D). Again, a positive correlation for IL-15 and a negative one for IL-7 was found with age in both groups, CMV^−^ and CMV^+^ persons. Interestingly, CMV^−^ persons clustered differently from CMV^+^ ones when IL-15 mRNA expression was studied in correlation with age.

Interactions with IL-15-producing cells in the BM are required for the survival of effector/memory CD8^+^ T cells (7, 8). To assess whether the increased expression of IL-15 in CMV^+^ persons affects the number of interactions between IL-15-producing cells and CD8^+^ T cells, we studied BM sections and quantified CD8^+^ T cells in close proximity to IL-15^+^ BM cells in CMV^−^ and CMV^+^ persons (Figure 5E). 13.7 ± 2.7 and 27.1 ± 5.2% of CD8^+^ T cells, respectively, were interacting with IL-15-producing cells in CMV^−^ and CMV^+^ persons (Figure 5F). In summary, our findings indicate that IL-15 expression increases not only with aging but also with CMV infection, and is highest in old CMV^+^ persons. Additionally, the increased production of IL-15 in the BM may attract more CD8^+^ T cells into the close proximity of IL-15-producing cells.

## Discussion

Latent CMV infection is currently believed to drive or at least exacerbate “immunosenescence” (37). Both conditions lead to a characteristic shift in the T cell repertoire with a decrease in naïve T cells and an increase in highly differentiated T cells, particularly within the CD8^+^ T cell subset (38). Numerous studies have, therefore, focused on the phenotype and function of these “terminally” differentiated CD8^+^ T cells (39), which are frequently CD28^−^. As a corresponding cell type does not exist in mice, most studies have been performed in human blood and lymphoid organs have very rarely been investigated (40).

The BM has recently been recognized for its important role in the maintenance of T cell memory, and the existence of particular niches for adaptive immune cells has been suggested, such as the IL-7 niche for the maintenance of CD4^+^ memory T cells (5). We demonstrated that, in the human BM, the production of IL-15 and the numbers of effector/memory CD8^+^ T cells increased with age and a link with inflammation was found (12, 32). However, it is still unclear how CMV positivity affects CD8^+^ T cells in the BM, particularly, highly differentiated effector cells. In previous studies, an increased frequency of effector/memory CD8^+^ T cells lacking the costimulatory molecule CD28 and expressing markers of T cell activation has been found in the BM in comparison to the PB (8, 32). The effects of aging on the production of BM survival factors for effector/memory T cells have recently been described (12). Thus, we were now interested in considering whether CMV may have an impact on the phenotype of effector CD8^+^ T cells not only in the PB but also in the BM in the context of the BM niches responsible for the maintenance of the immunological memory. Studies on CMV in old donors are frequently hampered by the fact that very few elderly persons are CMV^−^. We have now had the chance to analyze an interesting Austrian cohort all living in Upper Austria with an unusually high prevalence of CMV^−^ elderly donors. In addition, since only patients undergoing hip replacement surgery because of osteoarthrosis were included in the cohort, any possible effects of hip fracture on the immune system and the influence of depression frequently found in old patients with fractures could be excluded (41). Whether CMV infections are rare in this specific geographical area, or whether the low frequency of CMV infection is simply coincidence is not known. The availability of more than 30 BM samples from CMV^−^ persons enabled us to acquire interesting data on the comparison of BM T cells from CMV^+^ and CMV^−^ elderly persons. Thus, we could show that ${\text{T}}_{\text{EMRA}}^{\text{bright}}$ cells were more frequent in CMV^+^ persons than in age-matched CMV^−^ controls. CD45RA^+^ CCR7^−^ T cells have been shown to be a specific feature of CMV in the periphery (14), and we now know that this specific cell type can also be regarded as a marker of CMV infection in the BM. T_EMRA_ cells are frequently, but not always, CD28^−^ and KLRG-1^+^. It was of particular interest that CMV-specific changes of surface markers were observed in the CCR7^−^ CD45RA^bright^, but not in the CD45RA^dim^ population, suggesting that CMV does indeed drive T cell differentiation to its limits. ${\text{T}}_{\text{EMRA}}^{\text{bright}}$ cells were also more frequent in BM than in the PB (data not shown), indicating that this cell type is specifically attracted by the BM. In this context, it is of additional interest that KLRG-1^+^ IL-7Rα^−^ so-called short-lived effector cells (SLECs), which are also enriched in the ${\text{T}}_{\text{EMRA}}^{\text{bright}}$ subset, have been shown to be supported by IL-15 (42). As IL-15 production increases in the aged BM, we were interested in clarifying whether CMV infection had a similar effect. Indeed, we found that IL-15 production was higher in CMV^+^ compared to CMV^−^ donors (Figure 5A), however, both groups showed an increase in IL-15 production with age (Figure 5C). The highest IL-15 mRNA expression was in old CMV^+^ donors. The increased number of interactions between IL-15-producing cells and CD8^+^ T cells in CMV^+^ persons further supports our concept that the BM microenvironment in old age, in combination with CMV, strongly attracts and supports CD8^+^ T cells of a high differentiation status. The involvement of CMV in this process may be partly due to the fact that CMV is known to cause inflammation (15) and may, therefore, increase age-related inflammatory processes (43). In the BM, age-related changes such as the accumulation of reactive oxygen species (ROS) stimulate the production of IL-15, which in consequence attracts highly inflammatory T cells (12), resulting in a vicious circle, the results of which may be even more pronounced in CMV^+^ persons.

In contrast to aging *per se*, CMV does not seem to change the BM IL-7 niche, but may still be responsible for an imbalance between the production of IL-15 and IL-7. This imbalance could lead to a preferential accumulation of highly differentiated CD8^+^ T cells at the expense of CD4^+^ and CD8^+^ memory T cells and long-lived plasma cells.

In light of this possibility, our interest grew in the question whether the characteristic CD8^+^ T cell populations in the BM were able to respond to the obvious IL-15 overload in this organ in CMV^+^ persons. We, therefore, studied the common β-chain of the IL-15/IL-2 receptor (CD122) and found that this receptor was highly expressed in almost all CD8^+^ ${\text{T}}_{\text{EMRA}}^{\text{bright}}$ cells and was especially high in the CD28^−^ ${\text{T}}_{\text{EMRA}}^{\text{bright}}$ subset, which is particularly frequent in CMV^+^ persons. We have previously shown that IL-15 signaling takes place in the BM (8). Our present data specifically suggest that the combination of high IL-15 production and high CD122 expression most likely leads to pronounced IL-15 effects in BM CD8^+^ T cells of CMV^+^ persons.

Peripheral Ab concentrations against CMV correlated positively with the percentage of CD8^+^ CD28^−^ ${\text{T}}_{\text{EMRA}}^{\text{bright}}$ T cells in the BM, and there was a negative correlation between the peripheral Ab concentrations and IL-7Rα^+^ on ${\text{T}}_{\text{EMRA}}^{\text{bright}}$ T cells. The relationship was even more pronounced when a ratio between CD122 and IL-7Rα^+^ was used in the correlation. It has been shown that the humoral anti-CMV response is particularly high in advanced aging associated with comorbidity and cognitive and functional impairments (44). This is of interest but would be of no relevance for our study, as none of the participants had obvious cognitive problems or overt disease. We also did not see a significant correlation between CMV Ab titers in the serum and age (data not shown). Although it is unclear what very high anti-CMV IgG Ab concentrations mean in CMV^+^ clinically healthy persons, they may indicate active humoral defense against re-activation of the virus. CD8^+^ T cell responses may be of even greater relevance during re-activation.

Our data on the relationship between highly differentiated CD8^+^ T cells in the BM and peripheral Ab concentrations indicate that they may both be markers of an ongoing immune response against CMV.

In summary, our data suggest that latent CMV infection leads to changes in the BM, which disturb the balance among immunoregulatory processes in the BM, in particular between stromal cell niches and T cells. CMV infection may, therefore, be considered as a risk factor for deterioration of the immunological memory in the BM, particularly in elderly individuals.

## Ethics Statement

This study was carried out in accordance with the recommendations of the Ethics Committees of the “Klinikum Wels-Grieskirchen” (Austria) with written informed consent from all subjects in accordance with the Declaration of Helsinki prior to their inclusion in the study. The protocol was approved by the Ethics Committees of the “Klinikum Wels-Grieskirchen” (Austria).

## Author Contributions

LP and BG-L: study design, interpretation of data, critical appraisal, and final approval of the version to be published; KT: sample collection and study design; LP: method design; LP, EN, AM, BJ, and MK: experimental work.

## Conflict of Interest Statement

The authors declare that the research was conducted in the absence of any commercial or financial relationships that could be construed as a potential conflict of interest.

## Abbreviations

Ab, antibody; BM, bone marrow; BMMCs, BM mononuclear cells; CMV, cytomegalovirus; KLRG-1, killer cell lectin-like receptor G1; PB, peripheral blood; PBMCs, PB mononuclear cells; ROS, reactive oxygen species; SLECs, short-lived effector cells; T_EM_, effector memory T cells; T_EMRA_, terminally differentiated effector memory cells re-expressing CD45RA.
