# Supplementary figures and images for: Increased IL-15 Production and Accumulation of Highly Differentiated CD8+ Effector/Memory T Cells in the Bone Marrow of Persons with Cytomegalovirus
Source: Front Immunol. 2017 Jun 19;8:715. doi: 10.3389/fimmu.2017.00715 (PMC5474847; doi:10.3389/fimmu.2017.00715)

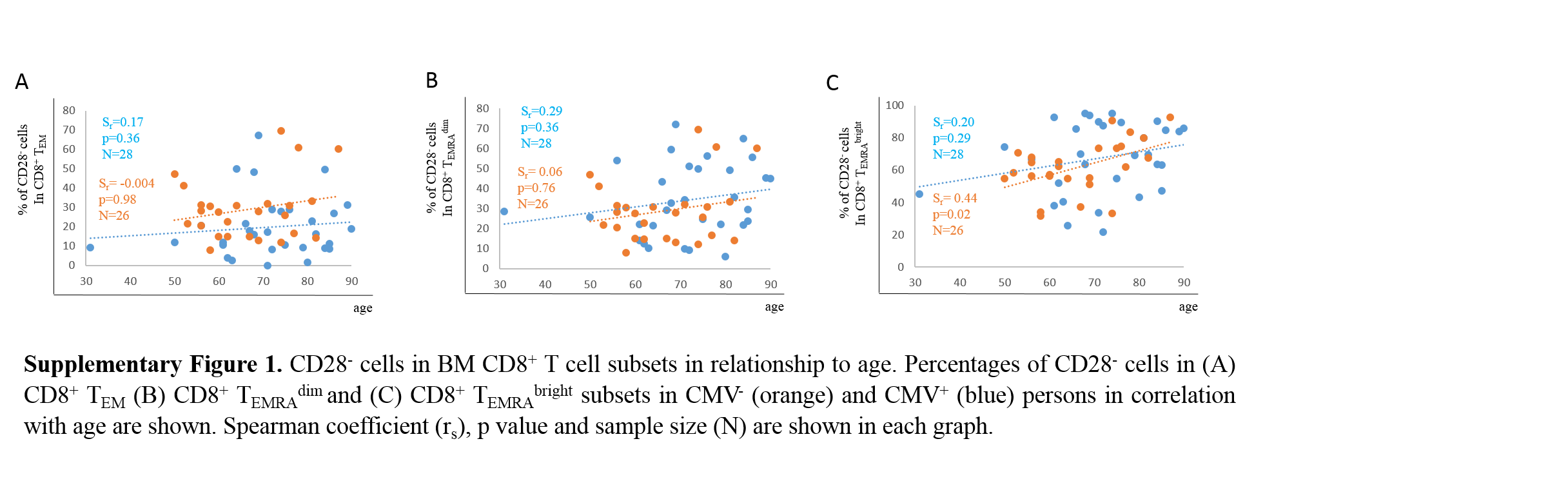

Supplement: Supplementary file 1 [file Image_1.TIF]

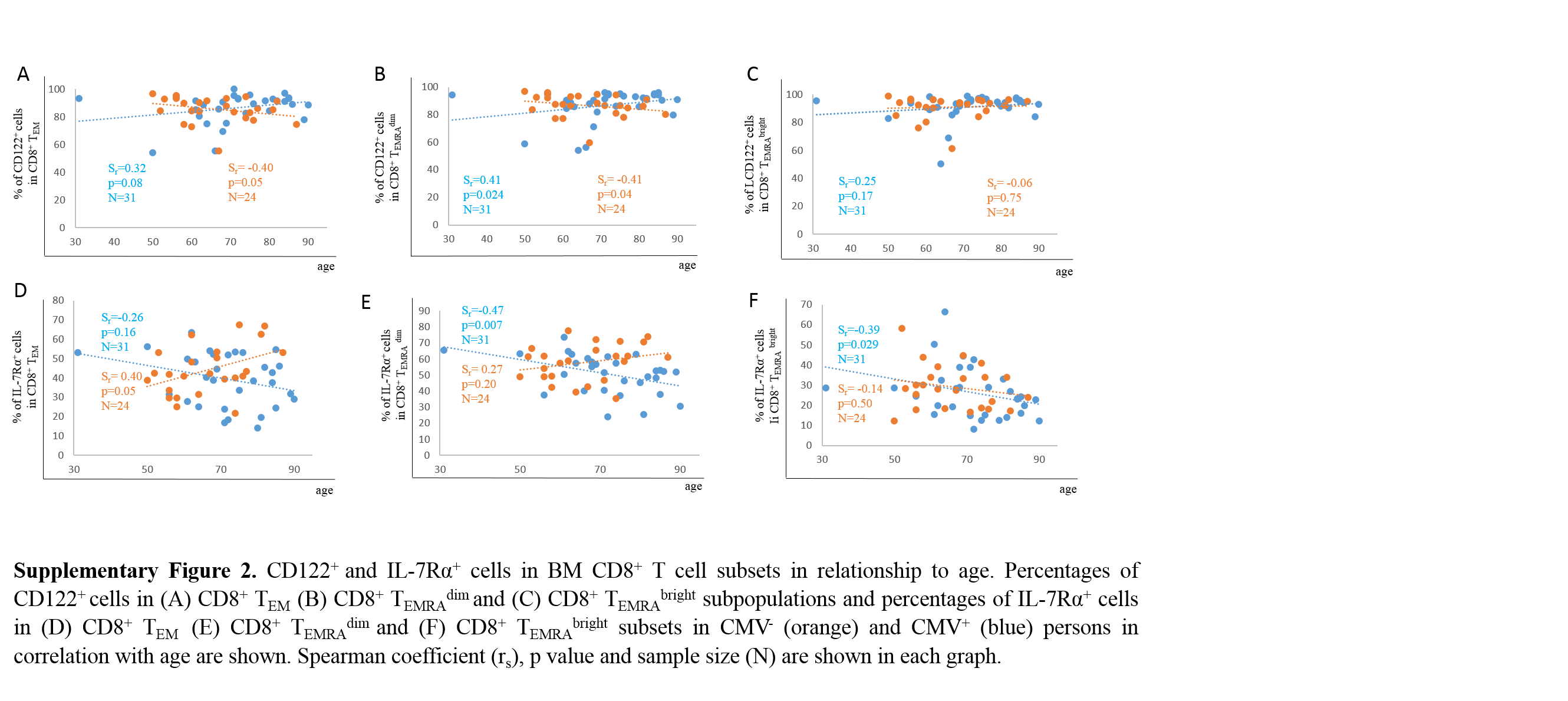

Supplement: Supplementary file 2 [file Image_2.TIF]
